# Supplementary material for: Defective neuronal differentiation in Lowe syndrome is associated with mitochondrial dysfunction and impaired cilia-related Sonic Hedgehog signaling
Source: eLife. 2026 Sep 18;13:RP104055. doi: 10.7554/eLife.104055 (PMC13588622; doi:10.7554/eLife.104055)

**Figure 3. Source Data 1. Genotyping processing for wild-type and IOB mouse.**

(a) Genotyping primers for wild-type and IOB mouse. (b) Genotyping processing for wild-type and IOB mouse.

**(a) Genotyping primers**

| **OCRL Wild-type (250 bp)** | **primer (5' to 3')** |
|---|---|
| GT-F | CCCTTTTCATCTGTTAGGAGAAAT |
| GT-R | GCATGGTTAAACGCACTATGTGG |
| **OCRL KO (480 bp)** | **primer (5' to 3')** |
| OCRL KO –F | GCCCTTTGATTCTAATCCCTTTTCATC |
| OCRL KO –R | TCTGAGCCCAGAAAGCGAAG |

| **Inpp5b (wt 150 bp/ mut 220 bp)** | **primer (5' to 3')** |
|---|---|
| E25-F | TAAAGTCTGAAAATCCAAGGC |
| E34-R | CTCATTTCTCCTTGATTCCAA |

| **INPP5B HumanBAC (240 bp)** | **primer (5' to 3')** |
|---|---|
| in Exon 1F | CCACCCCACGATTGACTC |
| in Exon 1R | GGTGTCCCAGCCCTCAG |

**(b) Genotyping processing**

Step 1 - OCRL WT

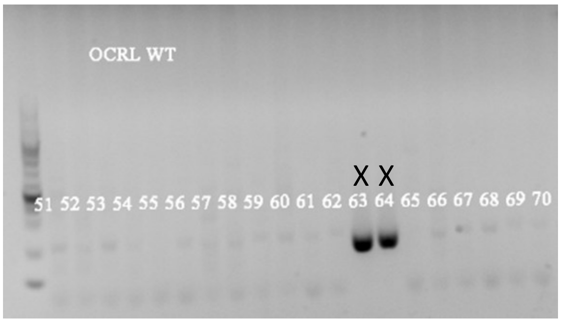


Step 2- OCRL KO

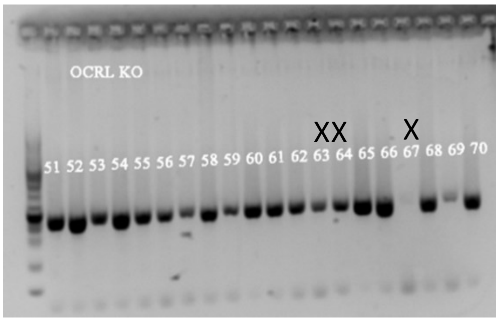


Step 3- Inpp5b (wt 150 bp/ mut 220 bp)

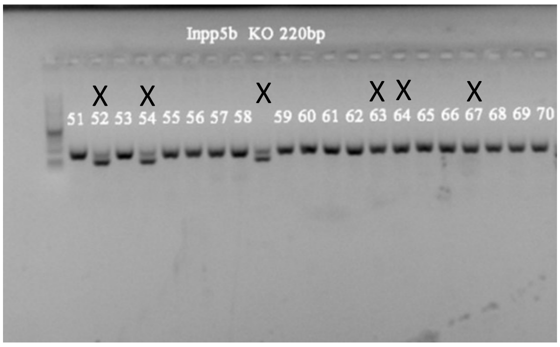


Step 4- INPP5B HumanBAC (240 bp)

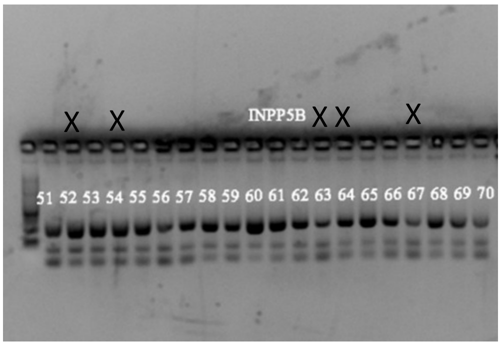

Supplement: Supplementary file 2 [file elife-104055-fig3-data1.zip › Figure 3—source data 1. PDF file containing genotyping processing for IOB mice/Figure 3 - source data 1.pdf]
