# Supplementary material for: Defective neuronal differentiation in Lowe syndrome is associated with mitochondrial dysfunction and impaired cilia-related Sonic Hedgehog signaling
Source: eLife. 2026 Sep 18;13:RP104055. doi: 10.7554/eLife.104055 (PMC13588622; doi:10.7554/eLife.104055)

Figure 5—source data 1. Validation of ocrl knockout zebrafish

a) Two guide RNAs (gRNAs) were designed to target the ocrl locus and co-injected with Cas9 into zebrafish embryos.

gRNA sequences:

gRNA1: 5’-TCTAACAAGGACAGGAGTCTTGG-3’

gRNA2: 5’-TCTGCGAGGTGAACGAACACCGG-3’

(b) Genotyping workflow used to distinguish wild-type and ocrl-KO alleles. Genomic DNA was extracted from larvae and amplified by PCR using the following primers:

Forward: 5’-AGAATGTGACACTGTTTCTC-3’

Reverse: 5’-GTGCAGGGAAAATAAGGTGT-3’

PCR products were analyzed to confirm successful genome editing at the target locus.

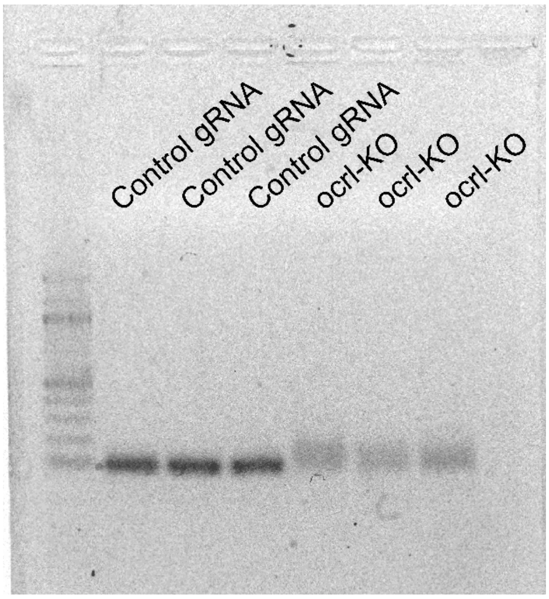

Supplement: Supplementary file 4 [file elife-104055-fig5-data1.zip › Figure 5—source data 1. PDF file containing the genotyping analysis of ocrl-KO zebrafish/Figure 5—source data 1.pdf]
