# Supplementary material for: Defective neuronal differentiation in Lowe syndrome is associated with mitochondrial dysfunction and impaired cilia-related Sonic Hedgehog signaling
Source: eLife. 2026 Sep 18;13:RP104055. doi: 10.7554/eLife.104055 (PMC13588622; doi:10.7554/eLife.104055)
Supplement: Peer review history

# Editor report: eLife Assessment

**DOI:** 10.7554/eLife.104055.3.sa0

**Authors:** Xiaorong Liu

**Affiliations:** University of Virginia United States

**Keywords:** Solid, Useful

This study investigated mitochondrial dysfunction and the impairment of the ciliary Sonic Hedgehog signaling in Lowe syndrome (LS), a timely topic given the limited research in this area. The data obtained from patient-derived iPSC neurons and a mouse model are **solid**. Although the main claims of the study are only partially supported by the current evidence, it provides a **useful** starting point for future functional studies investigating the link between mitochondrial defects and primary cilia in neural development.

# Referee report: Reviewer #2 (Public review)

**DOI:** 10.7554/eLife.104055.3.sa1

Summary:

This manuscript investigates how neural cell development is affected in Lowe syndrome. Using neural cultures differentiated from human iPSCs carrying either a LS mutation or a genetically engineered mutation in OCRL, the authors show a depletion of mitochondrial DNA and decrease in mitochondrial activities that correlate with an increased formation of astrocytes at the expense of neurons. Similar effects on mitochondria and on astrocyte development were observed in a LS mouse model. Moreover, these mutant brain cells are less likely to be ciliated and show a reduction in Sonic hedgehog signalling.

Strengths/Weaknesses:

The study derives strength from the analyses of two different models of Lowe syndrome, both reaching similar conclusions. However, the observed changes in mitochondrial defects, neuronal/astrocytic development and primary cilia are only correlated, with no attempt to investigate a causal relationship. Moreover, the mouse model is only analysed at the adult stage providing no insights into the development of the defects. Different brain regions are analysed with immunostainings and qRT-PCR making it challenging to draw clear correlations between these findings. The quality of the corresponding figures is often poor and the selection of markers is frequently inappropriate. Taken together, these limitations complicate the interpretations of the data and significantly limit the conclusions that can be drawn from the study.

Although the study remains incomplete as main claims are only partially supported it can be used as a starting point for future functional studies into the link between mitochondrial defects and primary cilia in neural development.

Comments on revised version:

I am afraid the revised manuscript does little to address the concerns I raised in my initial review. The authors have primarily revised the text, removed over-interpretations and discussed critical points as limitations of the study. This gives the impression that key concerns have merely been rationalised, particularly as only a few new experiments are presented. My main concerns therefore remain:

(1) The authors present three different phenotypes (altered neural differentiation, mitochondria dysfunction, alterations in primary cilia and ciliary Shh signalling) but a link between these phenotypes is not investigated. No mechanistic experiments are presented. Instead, the authors try to address the lack of a mechanism through refined wording but still use formulations that imply a direct link between these phenotypes. For example, their rebuttal letter finishes with the statement that the manuscript "provides a multi-model, cross-species framework linking mitochondrial dysfunction, ciliary signaling, and altered neural differentiation in Lowe syndrome". Similar formulations are used in the text.

(2) The authors still claim that ciliary Shh signalling is reduced but ignore the fact that Shh mRNA in iN cells and Shh protein in the IOB mouse are significantly decreased. This reduction represents the most likely explanation for the reduced levels of Gli1 and Ptc1 mRNAs (Shh target genes), rather than dysfunction of cilia. In order to test for cilia dysfunction, the authors need to use experiments in which they quantify the response of control and OCRL mutant cells to exogenously added Shh protein or Shh agonists. Moreover, the increased Gli1 protein expression in the IOB mouse contradicts the reduced levels of Gli1 mRNA.

(3) The analyses of the IOB mice are only done in 2 months old adult animals, nevertheless claims are made that changes in cell proportions are consequences of altered cell fate decisions. Alterations in proliferation and cell death are not addressed by experiments.

# Author comment: Author response

**DOI:** 10.7554/eLife.104055.3.sa2

**Authors:** Grzegorz Walkiewicz, Siyu Chen, Chienhui Lo, Jingyu Zhao, Zhiquan Liu, Biao Wang, Qing Wang, Tia J Kowal, Benjamin Lawson, Yang Sun

**Affiliations:** Stanford University School of Medicine Palo Alto United States; Stanford University School of Medicine Palo Alto United States; Stanford University School of Medicine Palo Alto United States; Stanford University School of Medicine Palo Alto United States; Stanford University School of Medicine Palo Alto United States; Stanford University School of Medicine Palo Alto United States; Stanford University School of Medicine Palo Alto United States; Stanford University School of Medicine Palo Alto United States; Stanford University School of Medicine Palo Alto United States; Stanford University School of Medicine Palo Alto United States

The following is the authors’ response to the original reviews.

**eLife Assessment**

This study investigated mitochondrial dysfunction and the impairment of the ciliary Sonic Hedgehog signaling in Lowe syndrome (LS), a timely topic given the limited research in this area. The data from patient iPSC-derived neurons and a mouse model were collected using solid methods, but the evidence supporting key claims is incomplete, and some technical aspects fall short of expectations. Despite these limitations, the study provides a useful foundation for exploring the relationship between mitochondrial defects and primary cilia in neural development.We appreciate the editorial assessment highlighting the importance of studying mitochondrial dysfunction and ciliary signaling in Lowe syndrome. We acknowledge that our study is largely associative, and we have revised the manuscript to clearly state this limitation, toned down causal claims, and emphasized that our work provides a foundation for future mechanistic studies.

We appreciate the editorial assessment highlighting the importance of studying mitochondrial dysfunction and ciliary signaling in Lowe syndrome. We acknowledge that our study is largely associative, and we have revised the manuscript to clearly state this limitation, toned down causal claims, and emphasized that our work provides a foundation for future mechanistic studies.

We have also:

- Improved figure clarity and consistency

- Corrected errors in gene annotations and normalization

- Refined the mechanistic framework linking OCRL, mitochondria, and cilia

New Experimental Data:

Figure 5, Supplementary Figure 4. We confirmed mitochondrial defects by generating *ocrl*-KO zebrafish (Supplementary Figure 4). We first assessed mitochondrial reactive oxygen species (mitoROS) using MitoSOX staining. Next, we evaluated mitochondrial membrane potential (ΔΨm) using MitoTracker CMXRos. Finally, we assessed mitochondrial content via TOM20 staining. For all analyses, we focused on the ocular and cranial regions of the zebrafish to maintain consistency (see Author response image 1). Notably, previous studies have reported that *ocrl*-KO zebrafish exhibit seizures and brain developmental abnormalities, supporting their relevance as a model for Lowe syndrome-like phenotypes [1].

**Author response image 1.** 

Figure 6 c. In addition to quantifying the proportion of ciliated cells in the IOB mouse brain, we measured cilia length and compared it between IOB and WT brain sections. Our results show that IOB mice exhibit elongated cilia compared to WT controls, suggesting that OCRL deficiency is associated with stress-related alterations in ciliary structure. These findings are consistent with previous studies reporting that cilia elongation can be associated with increased ROS levels and mitochondrial dysfunction [2,3].

**Public Reviews:**

**Reviewer #1 (Public review):**

The preparation of the manuscript requires improvement. There are many errors in the presentation of data.

We thank the reviewer for this important comment. We have carefully revised the manuscript to improve the clarity, accuracy, and consistency of data presentation.

Specifically, we have corrected inconsistencies in gene nomenclature (e.g., CO2 vs COX2, DLOOP) across the text, figures, and legends. We standardized normalization methods and ensured consistency between figures and descriptions. We revised figure labels, legends, and annotations for clarity and accuracy. We corrected referencing errors and ensured appropriate citation of prior work. We improved overall figure quality and readability. In addition, we performed a thorough review of the entire manuscript to eliminate typographical errors and ensure consistency in terminology and data interpretation.

The use of references needs to be re-considered. Sometimes a reference is used when in fact the results included in that paper are the opposite of what the authors intend.

We thank the reviewer for this important comment. We have carefully re-evaluated all references throughout the manuscript to ensure that they accurately reflect the findings they are cited to support. In cases where the cited studies did not fully align with our interpretation or could be misleading, we have either revised the text to more accurately represent the original findings or replaced the references with more appropriate sources. We have also clarified instances where prior studies report differing or context-dependent results to avoid overinterpretation.

The authors conclude the paper by claiming that mitochondrial dysfunction and impairments of the ciliary SHH contribute to abnormal neuronal differentiation in LS, but the mechanism by which this sequence of events might happen hasn't been shown.

We thank the reviewer for this important comment. We agree that the current study does not establish a direct causal mechanism linking mitochondrial dysfunction, ciliary SHH signaling, and altered neuronal differentiation in Lowe syndrome. Our data demonstrate that these processes co-occur consistently across multiple model systems, supporting a potential functional relationship. However, we acknowledge that the precise sequence of events and mechanistic connections remains to be defined. To address this, we have revised the manuscript to clarify that our conclusions are based on associative findings rather than direct mechanistic evidence. We have also updated the Discussion to explicitly acknowledge this limitation and to frame our model (Figure 7) as a proposed working hypothesis. Future studies will be required to determine whether mitochondrial dysfunction directly impacts ciliary SHH signaling and how these pathways influence neuronal differentiation.

Phenotype of increased astrocytes in both the IOB mouse brain or iPSC-derived cultures iN cells requires clarification as one of the markers used as an astrocyte marker, BRN2, is commonly used as a neuronal marker. As LS is a neurodevelopmental disorder, and the phenotype in question is related to differentiation, it is crucial to shed light on the developmental timeline in which this phenotype is seen in the mouse brain.

We thank the reviewer for this important comment. We agree that the use of BRN2 as an astrocytic marker was inappropriate, as it is primarily recognized as a neuronal marker. Accordingly, we have revised the manuscript to remove BRN2 from the interpretation of astrocytic identity and now rely on GFAP expression as the primary astrocytic marker. We have also clarified this point in both the Results and figure legends to avoid misinterpretation. In addition, we have revised the text to more accurately describe our findings as an altered balance in neuronal versus astrocytic marker expression, rather than a definitive increase in astrocyte numbers.

Regarding the developmental context, we acknowledge that Lowe syndrome is a neurodevelopmental disorder and that temporal aspects are highly relevant. In our study, the in vivo analyses were performed on adult 2-month-old IOB mouse brains, which we have now explicitly stated in the manuscript. We recognize that this limits our ability to directly assess developmental dynamics of lineage specification. We have therefore added this as a limitation in the Discussion and clarified that future studies examining earlier developmental stages will be necessary to determine when these alterations arise.

Mitochondrial dysfunction in astrocytes has been shown to induce a ciliogenic program. However, almost the opposite is shown in this paper, with regards to ciliation. Morphology of the cilia was not assessed either, which is an important feature of ciliary homeostasis. The improper ciliary homeostasis here appears to be the improper Shh signalling, which has not been shown to be related to mitochondrial dysfunction. This leaves one wondering how exactly the different phenotypes shown in this paper are connected.

We thank the reviewer for this important comment. We agree that the relationship between mitochondrial dysfunction, ciliogenesis, and Shh signaling is complex and not fully resolved in the current study.

As noted by the reviewer, prior studies have reported that mitochondrial dysfunction can promote a ciliogenic program [4]. In contrast, our data show a reduced proportion of ciliated cells together with increased cilia length, indicating altered ciliary homeostasis rather than a straightforward increase in ciliogenesis. To address this point, we have revised the manuscript to describe our findings as context-dependent alterations in ciliary parameters more clearly, and we now explicitly discuss this apparent discrepancy with the literature in the Discussion. We also acknowledge the reviewer’s point regarding ciliary morphology. In the revised manuscript, we have included quantification of cilia length in addition to the proportion of ciliated cells, and we have expanded the Methods section to detail how these measurements were performed. We agree that additional ultrastructural and functional analyses would further strengthen the characterization of ciliary homeostasis, and we now include this as a limitation and future direction.

Regarding the link between mitochondrial dysfunction, ciliary alterations, and Shh signaling, we agree that our study does not establish a direct mechanistic connection. Our data demonstrate that these phenotypes co-occur consistently across multiple models, but do not define causality. To address this concern, we have revised the manuscript to clarify that our conclusions are associative, and we now present our integrated model (Figure 7) as a working hypothesis rather than a demonstrated mechanism. We also explicitly state in the Discussion that future studies will be required to determine whether mitochondrial dysfunction directly impacts ciliary signaling and Shh pathway activity.

This paper lacks a clear mechanistic approach. While the data validates the 3 broad phenotypes mentioned, there is a lack of connection between these phenotypes or an answer to why these phenotypes appear. While the discussion attempts to shed light on this by referencing previous studies, some of the referenced studies show contradicting results. Hence, it would be beneficial to clarify these gaps with further experiments and address the larger question of the connection between the mitochondria, Shh signalling, and astrocyte formation.

We thank the reviewer for this important and insightful comment. We agree that the current study does not establish a direct mechanistic link connecting mitochondrial dysfunction, altered Shh signaling, and changes in neuronal versus astrocytic differentiation.

Our primary goal in this work was to identify and validate phenotypes associated with OCRL deficiency across multiple independent model systems. We demonstrate that mitochondrial dysfunction, oxidative stress, altered ciliary/Shh signaling, and changes in neural lineage-associated markers co-occur consistently in these models. However, we acknowledge that the causal relationships between these processes remain to be defined.

To address this concern, we have revised the manuscript to more clearly state that our conclusions are associative rather than mechanistic, and we now present our integrated model (Figure 7) as a working hypothesis that links these phenotypes through a potential mitochondria-ROS-signaling axis. We have also expanded the Discussion to explicitly acknowledge this limitation and to avoid overinterpretation of causality.

In addition, we have carefully re-evaluated and revised the cited literature to ensure accuracy, particularly in cases where prior studies report context-dependent or seemingly contradictory effects of mitochondrial dysfunction on ciliogenesis and signaling pathways. These points are now discussed more explicitly to better position our findings within the existing literature.

We agree that further experiments, such as targeted rescue of mitochondrial function or modulation of Shh signaling, will be necessary to establish causal relationships between these pathways. These directions are now clearly outlined in the revised Discussion as important next steps.

Most importantly, there is no mention of how the loss of OCRL, a 5-phosphatase enzyme, results in the appearance of the mentioned phenotypes. Since there are multiple studies in the field of Lowe Syndrome that shed light on the various functions of OCRL, both catalytic and non-catalytic, it is important to address the role of OCRL in resulting in these phenotypes.

We thank the reviewer for this important comment. We agree that the link between OCRL function and the observed phenotypes was not sufficiently developed in the original version of the manuscript.

In the revised manuscript, we have expanded the Discussion to more clearly outline how loss of OCRL could contribute to the observed mitochondrial, ciliary, and differentiation phenotypes. OCRL encodes a PI(4,5)P₂ 5-phosphatase that regulates phosphoinositide homeostasis and membrane dynamics. Disruption of this activity is known to affect endolysosomal trafficking, actin organization, and membrane remodeling-processes that are critical for organelle maintenance and ciliary function. We now discuss how these alterations could impact mitochondrial homeostasis, for example, through defects in membrane contact sites, vesicular trafficking, or organelle quality control pathways.

In addition, we have incorporated discussion of potential non-catalytic roles of OCRL, including protein–protein interactions and scaffolding functions, which may contribute to the coordination of intracellular trafficking and cytoskeletal organization. These aspects may provide an additional layer of regulation linking OCRL loss to both mitochondrial dysfunction and ciliary alterations.

We emphasize that, while these mechanisms are supported by prior studies, our data do not directly test them. Therefore, we have carefully framed this section as a plausible mechanistic framework rather than a demonstrated pathway and have explicitly stated this limitation. We also outline future experiments aimed at dissecting catalytic versus non-catalytic contributions of OCRL to these phenotypes.

There are numerous errors in the qPCR experiments performed concerning the genes that were assayed. The genes mentioned in the text section do not match those indicated in the graphs or legends. This takes away the confidence of the reader in this data.

We thank the reviewer for this important observation. We agree that the inconsistencies between the genes described in the text and those shown in the figures and legends could reduce confidence in the data. In the revised manuscript, we have carefully rechecked all qPCR experiments and corrected the gene names across the Results, figures, and figure legends to ensure full consistency. We have also standardized the nomenclature throughout the manuscript (including consistent use of gene symbols and formatting) and verified that all plotted data correspond to the correct targets.

In addition, we have clarified the qPCR methodology, including normalization (all data are normalized to GAPDH) and primer information, to improve transparency and reproducibility.

**Reviewer #2 (Public review):**

Summary:

This manuscript investigates how neural cell development is affected in Lowe syndrome. Using neural cultures differentiated from human iPSCs carrying either an LS mutation or a genetically engineered mutation in OCRL, the authors show a depletion of mitochondrial DNA and a decrease in mitochondrial activities that correlate with an increased formation of astrocytes at the expense of neurons. Similar effects on mitochondria and on astrocyte development were observed in an LS mouse model. Moreover, these mutant brain cells are less likely to be ciliated and show a reduction in Sonic Hedgehog signalling.

Strengths/Weaknesses:

The study derives strength from the analyses of two different models of Lowe syndrome, both reaching similar conclusions. However, the observed changes in mitochondrial defects, neuronal/astrocytic development, and primary cilia are only correlated, with no attempt to investigate a causal relationship. Moreover, the mouse model is only analysed at the adult stage providing no insights into the development of the defects. Different brain regions are analysed with immunostainings and qRT-PCR making it challenging to draw clear correlations between these findings. The quality of the corresponding figures is often poor and the selection of markers is frequently inappropriate. Taken together, these limitations complicate the interpretations of the data and significantly limit the conclusions that can be drawn from the study.

We have carefully revised the manuscript to address the concerns raised, and we have revised the manuscript with additional supporting data.

**Recommendations for the authors:**

**Reviewer #1 (Recommendations for the authors):**

The authors have checked the expression of neuronal markers NeuN and FoxG1, and apart from GFAP, they categorise Brn2 as one of the astrocytes markers that they have also checked. But Brn2 is not an astrocyte marker. It is a neuronal marker that is expressed in layer 2/3 of the cortex. In fact, Brn2 is reported to be a key driver of neurogenesis in primate telencephalon development1 and for reprogramming of astrocytes to neurons2. Hence, the only glial marker they have used here is GFAP. BRN2 is a neuronal marker. It has been used as a neuronal marker even in the reference (Zhang et al, 2013), from which the protocol for inducing iPSCs to induced neurons (iNs) was taken. Hence, the qPCR results in 1g of overexpression of BRN2 indicate an increase in expression of a neuronal marker, not an astrocyte marker.

We thank the reviewer for this important and well-founded comment. We fully agree that BRN2 is a neuronal marker and not an astrocytic marker, and that its inclusion as an astrocyte marker in our original interpretation was incorrect.

In the revised manuscript, we have removed BRN2 from the analysis and interpretation of astrocytic identity. We now treat BRN2 exclusively as a neuronal marker and have updated the Results, figure legends, and text accordingly. Specifically, the qPCR data previously presented in Figure 1g are now interpreted as reflecting neuronal marker expression, not astrocytic differentiation. We have also revised our conclusions to avoid overinterpretation of astrocyte abundance. Our findings are now described more accurately as an altered balance in neuronal versus astrocytic marker expression, rather than a definitive increase in astrocyte numbers. In this context, GFAP remains the primary astrocytic marker used in this study.

We acknowledge the reviewer’s point that reliance on a single astrocytic marker is a limitation. This has now been explicitly stated in the Discussion, and we note that additional astrocyte markers will be required in future studies to more comprehensively define lineage-specific changes.

Incorrect marker usage (BRN2 as astrocyte marker)

We thank the reviewer for identifying this critical issue. We corrected the classification of BRN2 as a neuronal marker. Also, we re-analyzed the interpretation accordingly, revised all relevant text and figures. Importantly, Astrocyte conclusions are now based primarily on GFAP expression, and we explicitly acknowledge this limitation in the Discussion

The graphs for the RT-PCR results indicate that gene expression values are normalized to actin whereas the legend mentions that they are normalized to GAPDH. This needs clarification.

We thank the reviewer for pointing out this inconsistency. We confirm that all qPCR data were normalized to GAPDH, and the reference to actin was an error. This has now been corrected throughout the figures, legends, and text to ensure consistency.

The use of wording to refer to the generation of induced neurons (iNs) should ideally be changed from "we developed"; as the protocol from Zhang et al, 2013 seems to have been directly adapted in this paper.

We thank the reviewer for this helpful suggestion. We agree that the wording was inappropriate. In the revised manuscript, we have replaced “we developed” with language indicating that iNs were generated using an established protocol, and we now explicitly state that the method was adapted from Zhang et al., 2013 [5].

OCRL KO iPSCs were obtained from Herbert Lachman's lab and not generated in this study. Hence, the Ran et al, 2013 reference is not necessary.

We thank the reviewer for this clarification. We agree that the OCRL knockout iPSCs were obtained from Herbert Lachman’s laboratory and were not generated in this study. Accordingly, we have removed the Ran et al., 2013 reference and revised the manuscript to clearly state the origin of the OCRL KO iPSC line.

The reference for Figure 1c is given as Ran et al, 2013 which is wrong. It should be Zhang et al, 2013.

We thank the reviewer for noting this error. We have corrected the reference for Figure 1c from Ran et al., 2013 to Zhang et al., 2013 in the revised manuscript.Limited in vivo mitochondrial characterization

Figure 1D, E: GFAP is a cytoskeletal marker but its expression here is very grainy and looks like an artifact. Is it possible to show the astrocyte phenotype using other astrocytes nuclei and cytosolic markers such as NFIA and S100B, respectively?

We thank the reviewer for this important suggestion. We acknowledge that GFAP is a cytoskeletal marker and that the signal in the current images may appear granular. We have carefully re-evaluated the staining and image processing to ensure that the signal represents true GFAP expression and have improved the image quality and presentation in the revised figures. We agree that inclusion of additional astrocytic markers such as NFIA and S100B would further strengthen the characterization. While we were not able to include these additional markers in the current revision, we now explicitly acknowledge this as a limitation in the Discussion and note that future studies will incorporate a broader panel of astrocyte markers to more comprehensively define astrocytic identity.

Since the authors have not used enough markers to understand the cell-type composition in WT and OCRL KO/mutant lines, it's not sufficient to conclude that the NPCs preferentially favour astrocytes over neuronal lineage. Any conclusive comments regarding the cell-state/cell-type specification necessitate evidence such as genetic lineage tracing using reporters for neuronal and astrocyte markers, and/or RNA/ATAC/scRNA sequencing.

We thank the reviewer for this important point. We agree that the current marker panel is not sufficient to definitively determine cell-type composition or to conclude preferential lineage specification.

In the revised manuscript, we have tempered our conclusions and now describe our findings as changes in neuronal versus astrocytic marker expression, rather than evidence of a shift in lineage fate. We also explicitly acknowledge this limitation in the Discussion. We agree that approaches such as genetic lineage tracing, reporter-based assays, and single-cell transcriptomic or epigenomic analyses (e.g., scRNA-seq or scATAC-seq) would be required to rigorously define cell-state transitions and lineage outcomes. These are important directions for future studies and are now highlighted in the revised manuscript.

Figure 2:

The mt-DNA gene CO2 was checked, not COX2. A typographical error in the written section, which does not match with the qPCR graph of the same.

We thank the reviewer for noting this inconsistency. We confirm that the gene analyzed was CO2, and the reference to COX2 in the text was incorrect. This has now been corrected throughout the manuscript to ensure consistency between the text, figures, and qPCR data.

The word "neurogenesis" is used very loosely throughout the paper. In the opinion of the reviewer, there is no evidence presented that there is a defect in neurogenesis in either of the models used in this paper.

We thank the reviewer for this important comment. We agree that the term “neurogenesis” was used too broadly and is not directly supported by our data. In the revised manuscript, we have removed or replaced this term where appropriate and now refer more precisely to changes in neuronal versus astrocytic marker expression.

Line 150: They say that they have examined the functional properties of mitochondria during neurogenesis but it would have been better to understand OXPHOS at various time points of neurogenesis to actually conclude reduced OXPHOS 'during neurogenesis'. Moreover, genes related to other pathways such as glycolysis could have been checked to understand the bioenergetics of LS patients. Also, oxidative stress could have been checked using more than one marker. Since they are trying to understand the functional role of mitochondrial defects during neurogenesis, they could have performed live imaging of mitochondrial potential during various stages of neurogenesis. Isolation of mitochondria from LS patients and transcriptomics/proteomics might provide further clues about mitochondrial defects.

We thank the reviewer for these thoughtful suggestions. We agree that our data do not capture mitochondrial function across multiple stages of neurogenesis. In the revised manuscript, we have modified the wording to avoid implying temporal analysis “during neurogenesis” and instead describe mitochondrial parameters in differentiated cells. To strengthen the study, we have included additional in vivo validation in the zebrafish model, where we assessed multiple mitochondrial readouts, including mitochondrial membrane potential (ΔΨm) using MitoTracker CMXRos, oxidative mitochondrial stress ROS (mitoROS) using MitoSOX staining, and mitochondrial content (TOM20), supporting mitochondrial dysfunction across systems.

Elevated astrocytic reaction during the differentiation of NSPCs in the Lowe syndrome (IOB) mouse model.

Title: What does astrocyte reaction mean? This term should not be used without clear evidence of reactive astrocytes being present in the model.

We thank the reviewer for this important comment. We agree that the term “astrocytic reaction” is not appropriate without specific evidence of reactive astrocytes. In the revised manuscript, we have removed this terminology and replaced it with more accurate wording, describing our findings as altered astrocytic marker expression. This change better reflects the data and avoids overinterpretation.

In 1a, no quantification of the mouse brain size is given. From the given images alone, there appears to be no obvious decrease in brain size between the WT and IOB mice. This contradicts the text which indicates that the IOB mouse brain is smaller.

We appreciate this important point. We have:

Removed claims regarding reduced brain size

Clarified that our analysis was limited to available sections and no definitive conclusion about global brain morphology can be made

Figure 3E: Why is the astrocyte to neuron ratio measured using a cytoskeletal marker for astrocytes, GFAP but a nuclear marker for neurons, NeuN? Ratios to measure the percentage or proportion of astrocytes to neurons can only be checked by markers of the same nature such as GFAP to MAP2 (neuronal cytoskeletal marker) or NFIA (astrocyte nuclear marker) to NeuN.

We thank the reviewer for this important point. We agree that comparing a cytoskeletal marker (GFAP) with a nuclear marker (NeuN) is not ideal for deriving cell-type ratios. In the revised manuscript, we have removed the astrocyte-to-neuron ratio analysis and now present these data as relative marker expression/signals rather than proportions. We have also clarified this limitation in the text and Discussion.

(3) Lack of clarity in the experiments performed on the mice brains. PAX6 is used here as a neuronal marker along with NeuN, a mature neuronal marker. This is misleading as PAX6 is rather a marker for neural stem/progenitor cells and not neurons. The age of the mice has also not been mentioned, which is crucial considering the different markers used to characterize the mouse brain as well as since the authors are indicating that there is an abnormal neurodevelopment in the IOB mouse during development. Again, BRN2 is used here as an astrocyte marker. However, it is a neuronal marker. Hence, the phenotype of increased astrocytes currently is held by GFAP expression alone. Another astrocyte marker should be used.

We thank the reviewer for these important points. We have revised the manuscript to correct marker interpretation, now describing PAX6 as a progenitor marker rather than neuronal, and BRN2 as a neuronal marker, removing it from astrocyte-related analysis. We have also explicitly stated the age of the mice (2 months) in the Methods and Results. In addition, we have tempered our conclusions, describing the data as changes in marker expression rather than definitive cell-type shifts, and we now acknowledge that reliance on GFAP as a single astrocytic marker is a limitation, which is discussed in the revised manuscript.

Figure 6: Increase in astrocytes, mitochondrial dysfunction, and ciliary Shh signalling are 3 phenotypes discussed in this study. However, no experiments were done to shed light on the mechanistic connection between these phenotypes. This is reflected in the abstract shown in Figure 6. There is no comment on the mechanism behind these phenotypes.

We thank the reviewer for this important comment. We agree that the current study does not establish a direct mechanistic link between mitochondrial dysfunction, altered ciliary Shh signaling, and changes in astrocytic markers. Our aim was to identify and validate these phenotypes across multiple models. In the revised manuscript, we have clarified that Figure 7 represents a proposed working model based on associative findings rather than a defined mechanism. We have also revised the Discussion to explicitly acknowledge this limitation and to outline future experiments required to establish causal relationships between these

**Reviewer #2 (Recommendations for the authors):**

The authors report interesting findings in two different experimental models but the manuscript would benefit significantly from an analysis of a potential causal relationship between different findings. They often mention neural stem cells or the neuron/glia switch but their analysis of the mouse mutant is restricted to the adult stage. A more consistent analysis of specific brain regions would also be beneficial.

We thank the reviewer for this constructive comment. We agree that establishing causal relationships between the observed phenotypes is an important next step. In the revised manuscript, we have clarified that our conclusions are based on associative findings and have expanded the Discussion to outline experimental strategies that could address causality in future studies. We also acknowledge that our in vivo analysis is restricted to adult (2-month-old) IOB mouse brains, which limits our ability to assess developmental dynamics such as neural stem cell behavior or neuron-glial transitions. This limitation is now explicitly stated in the Discussion. Finally, we agree that region-specific analysis would strengthen the study. Due to the availability of samples, our analysis was not systematically performed across defined brain regions. We now acknowledge this limitation and note that future studies focusing on specific regions (e.g., cortex, hippocampus) will be important to better understand the spatial aspects of the phenotype.

Figure 1: The authors only measured the expression of marker genes by qRT-PCR. This could reflect higher expression levels in individual cells rather than a change in the proportion of neurons and astrocytes. They need to determine the cell proportions of astrocytes and neurons in addition. Moreover, there is a poor marker choice. Loss of FOXG1 expression could indicate a loss of telencephalic identity. BRN2 is expressed by cortical neurons.

We thank the reviewer for this important comment. We agree that qPCR-based marker analysis does not directly reflect cell-type proportions and may instead represent changes in gene expression per cell. Accordingly, we have revised the manuscript to avoid conclusions about cell proportions and now describe the data as changes in marker expression. We have also corrected marker interpretation, removing BRN2 from astrocyte analysis and clarifying that FOXG1 reflects telencephalic identity. These limitations and the need for more comprehensive cell-type characterization are now acknowledged in the Discussion.

In Figure 2, the authors determine the properties of mitochondria and claim that functional mitochondrial activities are decreased during neurogenesis in mutant iN cells. They need to take into account that according to Figure 1 the proportion of neurons and astrocytes may be changed. Hence, the decreased mitochondrial activity may reflect a fundamental difference between neurons and astrocytes. The authors need to clearly distinguish between neurons and astrocytes in their analysis. Moreover, the use of the term neurogenesis is confusing. They are analysing the neuron-to-glial switch, not the formation of neurons.

We thank the reviewer for this important comment. We agree that differences in cell-type composition may influence mitochondrial measurements. In the revised manuscript, we have tempered our interpretation, describing these data as changes in mitochondrial parameters at the population level rather than neuron-specific effects. We also acknowledge this limitation in the Discussion and note that cell-type-specific analyses will be required in future studies. In addition, we have revised the terminology throughout the manuscript, removing the term “neurogenesis” and instead referring to changes in neuronal versus glial marker expression to more accurately reflect the scope of our analysis.

Figure 3: The authors claim that astrocyte numbers are elevated in the IOB mouse model, however, it seems as if the authors analysed late postnatal, potentially adult brains but no age of the brains is provided. Given the large time lag between the formation of astrocytes and their analysis, the increased number of astrocytes could be due to a number of processes including altered proliferation and cell death. The authors need to investigate the proportion of astrocytes and neurons closer to the neuron-to-glia switch. Cell fate experiments like the long-term application of BrdU would be much better suited and would provide mechanistic insights. Again, markers are not adequate to reach their conclusion. Pax6 is only expressed in a tiny subset of neurons, Brn2 on the other hand is not astrocyte-specific as it is expressed in cortical neurons as well. Moreover, qRT-PCR analyses were done in the cortex and hippocampus whereas the boxes in Figure 3D are located in the basal ganglia. It would be much more informative and provide better comparisons to perform gene expression analysis and cell counts in the same brain regions.

We thank the reviewer for these important and constructive comments. We agree that our analysis is limited by the use of adult (2-month-old) IOB mouse brains, which do not allow direct assessment of developmental processes such as the neuron-to-glia transition. We have now explicitly stated the age of the animals and clarified this limitation in the Discussion, including the possibility that changes in astrocytic markers may reflect processes such as proliferation or survival rather than lineage specification.

We also agree that our marker panel was insufficient for definitive conclusions. Accordingly, we have revised the manuscript to remove overinterpretation, corrected marker usage, and now describe the data as changes in marker expression rather than cell-type proportions. The need for more rigorous approaches, such as lineage tracing (e.g., BrdU) and expanded marker panels, is now acknowledged as a future direction. Finally, we thank the reviewer for pointing out the inconsistency in the brain regions analyzed. We have clarified the regions used for qPCR, and we now explicitly acknowledge this limitation, noting that future studies will aim to perform region-matched molecular and histological analyses for more accurate comparisons.

Experiments in Figure 4 assess "whether changes in mitochondrial activity are involved in the altered differentiation of stem cells and progenitor cells in the LS mouse model" in 3-month-old brain sections. The murine adult brain only contains a few neural stem cells in the SVZ and in the dentate gyrus. Instead, this analysis needed to be done at late embryonic/early postnatal stages to capture the neuronal/glial switch. In addition, RT-PCR and immunostainings should be performed in the same brain region as stated above.

We thank the reviewer for this important point. We agree that analysis in adult (2-month-old) brains does not capture developmental stages such as the neuron-glia transition. We have revised the manuscript to remove implications of developmental analysis and now describe these data as mitochondrial parameters in adult tissue, explicitly acknowledging this limitation in the Discussion. We also clarify the brain regions used for qPCR and immunostaining and note as a limitation that these were not fully matched; future studies will perform region-specific, developmentally timed analyses.

Figure 5: The authors examine a potential link between mitochondrial defects and primary cilia. Mutant iN cell cultures contain lower levels of SHH mRNA and show concomitantly lower expression of the SHH target genes GLI1 and PTCH1. The authors link this finding with a reduced proportion of ciliated cells but the reduced SHH signalling is most likely explained by the decreased SHH expression. The authors also limit their analysis of primary cilia to one brain region, but they should also include the cortex and hippocampus as these regions were used for their qRT-PCR analysis. SHH signalling acts as a switch to stop the proteolytic processing of GLI3 and to promote the formation of the GLI3 activator form. It is therefore important to determine the ratio of GLI3 repressor and GLI3 activator using western blots. The authors claim that they found defective cilia formation, but cilia are poorly characterised. Are there differences in intraflagellar transport, the formation of the transition zone, etc? Is ciliary length altered? The authors only make a correlative link between mitochondrial defects and cilia but present no experiments to investigate causation. They should at least discuss potential mechanisms which could explain defects in cilia.

We thank the reviewer for these insightful comments. We agree that reduced SHH pathway activity may be influenced by decreased SHH expression, and we have revised the text to avoid overattributing this effect to ciliary changes. Our conclusions are now framed as associative, not causal.

We have expanded our cilia analysis to include quantification of both the proportion of ciliated cells and cilia length, and clarified these methods in the manuscript. We also acknowledge that additional characterization (e.g., intraflagellar transport, transition zone structure, GLI3 activator/repressor ratios) would further strengthen the analysis, and we now include this as a limitation and future direction. Regarding regional analysis, we agree that broader brain region coverage would be valuable. Due to sample availability, our analysis was limited, and this is now explicitly acknowledged as a limitation, with future studies aimed at region-matched analyses (e.g., cortex and hippocampus). Finally, we have expanded the Discussion to outline potential mechanisms linking mitochondrial dysfunction and ciliary alterations, while clearly stating that causal relationships remain to be established.

(1) The methods section does not contain any information on how immunostainings on brain sections were performed.

We agree that the description of immunostaining on brain sections was missing. We have now added a detailed protocol for brain section immunostaining in the Methods section to improve clarity and reproducibility.

(2) The abbreviation "RT-PCR" is used for both, real-time PCR and reverse transcription PCR

We also acknowledge the inconsistent use of the term “RT-PCR.” In the revised manuscript, we have standardized the terminology, using “qPCR” (quantitative real-time PCR) throughout to avoid confusion

Conclusion

We believe that these revisions significantly strengthen the manuscript. While the study remains primarily associative, it provides a multi-model, cross-species framework linking mitochondrial dysfunction, ciliary signaling, and altered neural differentiation in Lowe syndrome.

References:

(1) Ramirez IB-R, Pietka G, Jones DR, Divecha N, Alia A, Baraban SC, et al. Impaired neural development in a zebrafish model for Lowe syndrome. Hum Mol Genet. 2012;21:1744–59. https://doi.org/10.1093/hmg/ddr608

(2) Kim JI, Kim J, Jang H-S, Noh MR, Lipschutz JH, Park KM. Reduction of oxidative stress during recovery accelerates normalization of primary cilia length that is altered after ischemic injury in murine kidneys. Am J Physiol Renal Physiol. 2013;304:F1283-1294. https://doi.org/10.1152/ajprenal.00427.2012

(3) Moruzzi N, Valladolid-Acebes I, Kannabiran SA, Bulgaro S, Burtscher I, Leibiger B, et al. Mitochondrial impairment and intracellular reactive oxygen species alter primary cilia morphology. Life Sci Alliance. 2022;5:e202201505. https://doi.org/10.26508/lsa.202201505

(4) Ignatenko O, Malinen S, Rybas S, Vihinen H, Nikkanen J, Kononov A, et al. Mitochondrial dysfunction compromises ciliary homeostasis in astrocytes. J Cell Biol. 2022;222:e202203019. https://doi.org/10.1083/jcb.202203019

(5) Zhang Y, Pak C, Han Y, Ahlenius H, Zhang Z, Chanda S, et al. Rapid Single-Step Induction of Functional Neurons from Human Pluripotent Stem Cells. Neuron. 2013;78:785–98. https://doi.org/10.1016/j.neuron.2013.05.029
